# Exosomal MicroRNAs Mediating Crosstalk Between Cancer Cells With Cancer-Associated Fibroblasts and Tumor-Associated Macrophages in the Tumor Microenvironment

**DOI:** 10.3389/fonc.2021.631703

**Published:** 2021-04-01

**Authors:** Tong Su, Panpan Zhang, Fujun Zhao, Shu Zhang

**Affiliations:** ^1^Shanghai Key Laboratory of Gynecology Oncology, Department of Gynecology and Obstetrics, Renji Hospital, Shanghai Jiao Tong University School of Medicine, Shanghai, China; ^2^Department of Urology, Shanghai General Hospital, Shanghai Jiao Tong University School of Medicine, Shanghai, China

**Keywords:** tumor microenvironment, cancer-associated fibroblasts, tumor-associated macrophages, exosomes, microRNA

## Abstract

Exosomes are small extracellular vesicles containing diverse bioactive molecules. They play essential roles in mediating bidirectional interplay between cancer and stromal cells. Specific elements are selected into different types of exosomes via various mechanisms, including microRNAs (miRNAs), a subset of non-coding RNA that could epigenetically reprogram cells and modulate their activities. Cancer-associated fibroblasts (CAFs) and tumor-associated macrophages (TAMs) are two major types of stromal cells inhibiting immune response and facilitating tumor progression. Notably, accumulated studies provided critical evidence regarding the significance of exosomal miRNA–mediated intercellular crosstalk between cancer cells with TAMs and CAFs for tumor progression. This review aimed to summarize the current knowledge of cell–cell interactions between stromal and cancer cells conveyed by exosome-derived miRNAs. The findings might help find effective therapeutic targets of cancer.

## Introduction

Tumor microenvironment (TME) is a heterogeneous population of cells, which plays an important role in tumor progression and metastasis. It comprises many non-tumor host cells called stromal cells, including tumor-associated macrophages (TAMs), cancer-associated fibroblasts (CAFs), regulatory T cells, myeloid-derived suppressor cells, endothelial cells, pericytes, and platelets (1). Further, the extracellular matrix component and exosomes also comprise TME (1). TAMs and CAFs are two major components. Macrophages are the predominant inflammatory cells; when they dwell in TME, they are called TAMs. TAMs are the main leukocytes infiltrating solid tumors. They are largely derived from circulating monocytes (2) and are typically of M2-like phenotype (3). They have been found to favor malignant progression via diverse mechanisms. Additionally, CAFs are a specialized group of fibroblasts with heterogeneous origin and phenotypes (4). They produce large amounts of materials, such as extracellular matrix, chemokines, cytokines, and pro-angiogenesis factors, and facilitate tumor progression.

**Figure 1:**
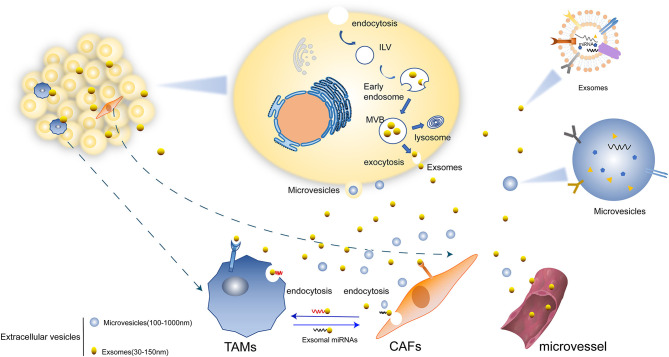
Mechanisms of EVs secretion. (1) Microvesicles (MVs) are formed directly from the budding of plasma membrane. (2) EVs can transform firstly as intraluminal vesicles (ILVs) within the lumen of multivesicular endosomes (MVBs) and be secreted upon fusion of the plasma membrane which lastly form exosomes. (3) Exosomes mediated the crosstalk between tumor cells with TAMs and CAFs. There are miRNAs and other molecules in exosomes and are transferred in to various cells. Some exosomes are transported through micro vessel and others could be directly internalized by TAMs, CAFs, and other cells.

Substantial crosstalk exists between tumor and stromal cells, especially TAMs and CAFs (5, 6). A large number of molecules in TME and specific TME stimuli boost the recruitment and activate TAMs and CAFs, such as tumor growth factor-β (TGF-β), interlukin-6 (IL-6), various types of growth factors, and cytokines, DNA fragments, as well as coding and non-coding RNAs (7). Besides, TAMs and CAFs also release molecules that participate in promoting tumorigenesis, cell proliferation, angiogenesis, and drug resistance. Most of the recent studies focus on the exosome-mediated cell–cell interaction (Figure 1).

### Exosomes

Extracellular vesicles (EVs) are a kind of vesicle structure with membrane structure released from cells, which have lipid bilayer-enclosed extracellular structures (8). EVs mainly include microvesicles (MVs) (100–1,000 nm) and exosomes (30–150 nm) according to the biosynthesis or release pathway. EVs had been used to dispose harmful or useless intracellular components during the cell development. A growning studies have found that EVs are essential intercellular communication vesicles which involve in various physiological and pathological processes. For example, EVs can regulate the homeostasis of TME by targeting CAFs, endothelia and immune cells and changing the structure and composition of the extracellular matrix (ECM) to promote tumor progression including proliferation, angiogenesis, migration, metastasis, immune regulation, and so on (9).

MVs were directly observed to bud from the plasma membrane in many types of cells, including endothelial cells, platelets, erythrocytes and cancer cells (10). MVs can carry proteins, lipids and nucleic acids expressed in abovementioned cells to involve in coagulation, immunomodulation, angiogenesis and initiate apoptosis, which is important for the progression of atherosclerosis, coronary heart disease and cancer (11).

Exosomes are nanosized membrane vesicles secreted from the intracellular multivesicular bodies (MVBs) or late endosomes via exocytosis into extracellular space (12, 13), which is different from the release pathway of MVs. The term “exosomes” was coined in 1981 by Trams et al. and referred to as “exfoliated membrane vesicles with 5′-nucleotidase activity” (14). Canonically, exosomes are dismantled by lysosomes and are also originally considered to be part of a cellular waste pathway (15). With further exploration, researchers discovered numerous robust and exciting functions. One is acting as vital carriers loaded with a plethora of macromolecular cargo, including proteins, lipids, coding RNA (mRNA), as well as all sorts of non-coding RNAs. It mediates the communication between cells (16). The effects of exosomes on different recipient cells are different due to their varied kinds of cell surface receptors. The role of exosomes and their contents in tumor growth and development is complex and multifaceted. For example, they are involved in immune responses by activating regulatory T cells and decreasing the function of CD8^+^ cell and natural killer (NK) cells (17). A recent study revealed that TEXs could participate the immune response to promote tumorigenesis via modulating angiogenesis and promoting epithelial-to-mesenchymal transition (EMT) and metastasis (18, 19) which is important to promote tumor progression and metastatic dissemination.

### miRNA

MicroRNA (miRNA) is endogenously expressed, small, single-stranded, non-coding RNA with typically 19–25 nucleotides (20). Two types of RNase, including RNase Drosha and Dicer, and RNA-induced silencing complex are needed in the maturation and activation of miRNA (21). Then, activated miRNA bind to the 3′-untranslated region of target messenger RNA (mRNA), acting as a transcription inhibitor either via direct repression of targeted mRNA or mRNA cleavage, and downregulate protein expression epigenetically. MiRNA plays a fundamental role in various biological processes, such as tissue differentiation, cell proliferation (22). Abnormal miRNA expression is associated with a variety of human diseases, such as inflammatory diseases and cancer. For instance, dysregulation of miR-144 was involved in the prostate cancer growth and metastasis via regulating the expression of EZH2 (23). Another study revealed that miR-21-5p upregulated MAPK/ERK signaling and promote the proliferation of leukemic B cells (24). Hence, miRNAs play important roles in cancer development, including invasion, metastasis, and drug resistance, and could be regarded as potential non-invasive biomarkers in cancer diagnosis and treatment (25).

### miRNA in Exosomes in Tumor Microenvironment

Exosomes in TME transfer various types of materials into the extracellular milieu, leading to the distinct functional changes in tumor and stromal cells. One major component is miRNAs, which was transferred to recipient cells to play a role in changing cellular behavior (26). Exosomes are secreted by all types of cells (27). MiRNA delivered by exosomes can regulate mRNA levels in recipient cells to block translation (28). However, the contents are specific and heterogeneous. Evidence shows that both the surface markers and the cargo vary in different types of cells and demonstrate specific exosome signatures (29). This indicates that miRNAs may be incorporated into exosomes selectively. Villarroya-Beltri et al. suggested that the protein heterogeneous nuclear ribonucleoprotein A2B1 (hnRNPA2B1) participated in this process via binding exosomal miRNAs specifically through the recognition of these motifs and controlling their loading into exosomes (30). The role of hnRNPA2B1 in the recruitment of RNA into exosomes was also identified in HEp-2 or HEK293T cells (31). Other mechanisms involved are still being explored. Further, human papillomavirus 16 E6 and E7 oncoprotein expression causes changes in microRNAs excreted in exosomes, and the miRNAs change mostly parallel those observed intracellularly (30). The exosomal miRNAs dysregulation can influence the crosstalk between cancer cells and tumor stromal cells (32). The miRNAs are released into the TME and target specific cells, promoting angiogenesis, increasing the infiltration of TAMs and CAFs, reprogramming the immunity to promote tumor progression, and so forth. MiRNAs are identified to have a double face in tumor modulation (33). The miRNAs in TEXs, however, are widely acknowledged as promoting factors in tumor progression, metastasis, and chemoresistance. Here, we discuss the interaction regulated by exosomal miRNAs in tumor microenvironment.

## Crosstalk Between Tumor Cells and CAFs Via Exosomal Mirnas

### CAFs

CAFs are major tumor stromal components in TME in solid tumors (34). Recent research shows the abnormal activation of CAFs may be due to the uncontrolled epigenetic control of gene expression and metabolic adaptive response (35). Activated CAFs alter cell surface markers are different from normal fibroblasts (NFs) (36). CAFs have both pro-tumorigenic and anti- functions, which are likely dynamic during tumorigenesis. For example, CAFs can promote many aspects of tumor progression including tumor growth, EMT, metabolism, invasion and metastasis (34). Further, CAF can exert its tumorigenic function directly (intercellular communication) and indirectly (soluble factors, miRNA). Erdogan et al. suggested that direct communication of CAFs with tumor cells and other stromal cells, such as endothelial and immune cells, in turn induces the acquisition of a specific biological phenotype of CAFs (37). Exosomal miRNAs have been identified as a crucial mediator in this intercellular crosstalk (Table 1).

**Table 1:** Exosomal miRNAs mediating the communication between tumor cells and CAFs.

**Origin**	**miRNAs**	**Donor (Markers)**	**Receptor (Markers)**	**Target and pathway**	**Functional change**	**References**
Tumor cells	miR-105	BC	CAFs	MYC signaling pathway	Inducing a metabolic program in cancer-associated fibroblasts (CAFs)	(38)
	miR-122	BC	CAFs	Glycolytic enzyme pyruvate kinase	Suppressing glucose uptake in lung fibroblasts; reducing primary tumor growth while enhancing metastasis	(39)
	miR-21/-146a	CLL	CAFs (α-SMA)	AKT/ERK/NF-κB signaling pathway	CAF induction, MSC proliferation, and EC angiogenic activities	(40)
	miR-10b	CRC	CAFs (α-SMA)	PIK3CA/PI3K/Akt/mTOR signaling pathway	CAF induction	(41)
	miR-21	HCC	CAFs (α-SMA, FAP, FSP)	PTEN/pyruvate dehydrogenase kinase 1 (PDK1)/Akt	Promoting angiogenesis; expression of VEGF, MMP2, MMP9, bFGF, and TGF-β	(42)
	miR-1247-3p	HCC	CAFs (α-SMA)	B4GALT3/β1-integrin/NF-κB signaling pathway	CAF activation; release pro-inflammatory cytokines such as IL-6 and IL-8 to foster metastasis	(43)
	miR-27a	GC	CAFs (α-SMA)	mediator complex subunit 14 (CSRP)	CAF induction; activate oncogenes, promote proliferation, motility, and metastasis	(44)
	miR-142-3p	LAC	CAFs (α-SMA, PDGFR-β)	TGFβR1	CAF tumor proliferation induction; promote angiogenesis and	(45)
	miR-155-5p	Melanoma	CAFs (α-SMA, FAP)	Activates SOCS1/JAK2/STAT3 signaling pathway	Proangiogenic switch of CAFs: expression of VEGFa, FGF2, and MMP9	(46)
	miR-211	Melanoma	CAFs (FSP-1)	IGF2R/MAPK signaling pathway	CAF induction	(47)
	miR-21-5p	OSCC	CAFs (α-SMA, vimentin)	mTOR/PI3K, STAT3/β-catenin signaling pathway	cisplatin (CDDP) resistance.	(48)
	miR-21/-378e/-143	CAFs (–)	BC	–	EMT	(49)
	miR-9	CAFs (α-SMA)	BC	E-cadherin	Metastasis, NF biological behavior	(50)
	miR-15a	CAFs (α-SMA, vimentin)	CCA	Plasminogen activator inhibitor 2 (PAI2)	Cell metastasis	(51)
	miR-21	CAFs (FSP1, α-SMA vimentin)	CRC	reversion-inducing-cysteine-rich protein with Kazal motifs (RECK)/MMP2	Cell proliferation, epithelial invasiveness, and oxaliplatin resistance	(52)
	miR-21	CAFs (α-SMA, fibronectin ED-A, paladin, and vimentin)	CRC	Extracellular signal-regulated kinases (ERK) and Akt	Progression and metastases	(53)
	miR-31	CAFs (α-SMA, FAP, vimentin)	CRC	Beclin-1, ATG, DRAM, and LC3	Proliferation, invasion, apoptosis, and radiosensitivity	(54)
	miR-148b	CAFs (α-SMA, vimentin)	EMC	DNA methyltransferase 1 (DNMT1)	Promoting invasion and cancer metastasis	(55)
	miR-21	CAFs (periostin, α-SMA, and podoplanin)	GC	PTEN/PI3K/Akt signaling pathway	Cisplatin resistance	(56)
	miR-320a	CAFs (a-SMA, FAP)	HCC	Pre-B cell leukemia homeobox 3 (PBX3)/MAPK pathway	Cell proliferation and metastasis (EMT, expression of CDK2, and MMP2)	(57)
	miR-335	CAFs α-SMA, FAP, FSP1)	HCC	Cell division cycle 42 (CDC42) and CDK2	Proliferation and invasiveness	(58)
	miR-7	CAFs (α-SMA, FAP, FSP1)	HNC	RAS-association domain family 2 (RASSF2)	Decreasing the secretion of PAR-4 from CAFs and enhancing proliferation and migration	(59)
	miR-196a	CAFs (α-SMA, FAP, FSP1)	HNC	cyclin-dependent kinase inhibitor 1B(CDKN1B) and inhibitor of growth 5 (ING5)	Cisplatin resistance	(60)
	miR-1	CAFs (α-SMA, FAP)	LAC	CXCR4-mediated signaling pathway, which involved NF-κB, and Bcl-xL	Proliferation and chemoresistance	(61)
	miR-21	CAFs (periostin, α-SMA, and podoplanin)	LAC	–	Poor prognosis	(62)
	miR-21	CAFs (α-SMA)	OC	Apoptotic protease activating factor-1 (APAF1)	Paclitaxel resistance	(63)
	miR-34a-5p	CAFs (α-SMA)	OSCC	AKT/GSK-3β/β-catenin/Snail signaling	proliferation and motility of OSCC	(64)
	miR-1228	CAFs (α-SMA)	OS	Downregulating endogenous SCAI	Cell migration and invasion	(65)
	miR-409-3p/-5p	CAFs (–)	PC	Ras suppressor 1 and stromal antigen 2	Facilitating tumorigenesis, epithelial-to-mesenchymal transition (EMT), and bone metastasis	(66, 67)

### Effect of Exosomal miRNAs Released by Cancer Cells on CAFs

The cancer cells tend to release more exosomes into TME and confer specific phenotypes and characteristics to stromal cells, which endow stromal cells with the pro-tumor phenotype and thus facilitate tumor progression. Finding of exosomal miRNAs provides novel insights on the formation and activation of CAFs. CAFs are of heterogeneous origin and originate from (1) Mesenchymal Stem Cells (MSCs); (2) bone marrow cells; (3) Pericytes and endothelial cells (ECs); (4) Resident quiescent normal fibrolasts (36). Increasing evidence confirms that the exosomal miRNAs derived from various tumor cells mediate this process. MSCs isolated from different tissues are one of the important sources of CAFs. MiRNA of tumor exosomes can interact with MSCs in TME, which can transform MSCs into CAFs. MiR-21 and miR-146a were the most abundant miRNAs known as critical regulators of CAF induction, involved in this process (68, 69). In addition, endothelial cells (ECs) can be induced by TGF-β and converted into CAFs through EMT, and exosomal miR-21-5p promote this process through activating TGF-β/Smad pathway in gastric cancer (GC) (64). Recent report has shown the abundant miRNAs in TEXs might transform NFs into CAFs to promote tumor survival. Melanoma miRNAs can promote the activation of NFs to transform into CAF, which can regulate lots of genes to induce significant changes in CAF gene expression (70). Chronic lymphocytic leukemia (CLL)–derived exosomes internalized by stromal cells delivered functional microRNAs and proteins and induced a CAFs-like phenotype in the target cells. An innovative study showed that colorectal cancer (CRC) cells released exosomes with a high level of miR-10b compared with normal cells, acting as a potential promoter for transformation. The significantly suppressed PIK3CA expression and decreased phosphatidylinositol 3-kinase/AKT/mammalian target of rapamycin (PI3K/AKT/mTOR) pathway activity were involved in this process (41). The high level of miR-27a in exosomes released from GC cells was suggested to induce the reprogramming of fibroblasts into CAFs as well as activate oncogene and promote proliferation, motility, and metastasis of cancer cells (44). Lawson et al. reported that exosomal miR-142-3p, a tumor suppressor in lung adenocarcinoma cells (LAC), triggered CAF phenotype independent of TGFβ signaling. When internalized by ECs, it also promoted angiogenesis through the inhibition of TGFβR1 (45). Another critical role of the interaction between tumor-derived materials and CAFs in TME is facilitating cancer metastasis. Mesothelial-to-mesenchymal transition (MMT) (71, 72) and epithelial-to-mesenchymal transition (EMT) (73) might be involved in this process. For example, exosomal miR-21 induces MMT in the peritoneal cavity to promote cancer dissemination in lung cancer (74) Mesothelial cells were identified as a source of CAFs in peritoneal carcinomatosis (72). Li et al. found that GC-derived exosomal miR-21-5p could lead to MMT of peritoneal mesothelial cells (PMCs) and activation of CAFs through TGF-β/Smad pathway by targeting SMAD7 *in vivo* (64). In addition, the expression of exosomal miR-106b-3p was significantly higher in colorectal cancer (CRC) patients with metastasis. Further, low-metastatic CRC cells were cocultured with high-metastatic CRC cell-derived miR-106b-3p can promote these cells EMT and metastasis *in vitro* (75). Exosomal miR-1247-3p was capable of inducing CAF activation by targeting β-1,4-Galactosyltransferase III (B4GALT3) and activating β1-integrin nuclear factor-κB (NF-κB) signaling, thereby releasing pro-inflammatory cytokines such as IL-6 and IL-8 to foster lung metastasis of liver cancer (43). Further, melanosomal microRNA-211 transferred into primary fibroblasts directly targeted insulin like growth factor 2 receptor (IGF2R) and activated mitogen-activated protein kinase (MAPK) signaling, thus triggering changes and reciprocally encouraging melanoma growth and invasion (47).

Angiogenesis is a vital part involved in both progression and metastasis. Tumor is an aggregation of heterogeneous cells with a higher requirement of nutrients and oxygen and the ability to evacuate metabolic wastes and carbon dioxide (76). Thus, the generation of neovasculature plays a crucial role. Studies show that hypoxia is a strong inducer (77). Increasing number of studies explored the relationship between CAFs and vascularization, and various mechanisms have been identified. A part of the relationship was mediated by exosomal miRNAs. Melanoma cell–secreted exosomal miR-155-5p activated the suppressor of cytokine signaling 1/janus kinase 2/signal transduction and activator of transcription 3 (SOCS1/JAK2/STAT3) signaling pathway and induced the proangiogenic switch of CAFs. The altered CAFs elevated the expression levels of proangiogenic factors, including vascular endothelial growth factor A (VEGFA), fibroblast growth factor 2(FGF2), and matrix metallopeptidase 9 (MMP9) (46). Further, the exosomal miRNA-21 converted hepatocyte stellate cells into activated CAFs which secrete angiogenic cytokines, including VEGF, MMP2, MMP9, bFGF, and TGF-β, thus promoting angiogenesis (42). MiRNAs in CAFs also enhanced the formation of vessels and increased the microvessel density reciprocally. Additionally, the extracellular vesicle secretion of miR-142-3p from LAC could also be transferred to endothelial cells, thus promoting angiogenesis through the inhibition of TGFβR1 (45).

Recently, more attention has been paid to the metabolic reprogramming of CAFs. Many types of metabolic adaptations have been described, including the Warburg effect, use of glutamine, and “reverse Warburg effect,” which introduce the metabolic interactions between tumor cells and CAFs (78). Studies assessing this have been performed for several years, but the available data remain insufficient. Yan et al. found that extracellular-vesicle-encapsulated miR-105 secreted by breast cancer cells was capable of inducing a metabolic program in CAFs: (1) enhancing glucose and glutamine metabolism to supply adjacent cancer cells; (2) accelerating elimination of wastes and detoxifying metabolic wastes, with the conversion mediated by activating MYC signaling in CAFs (38); and (3) biasing the nutrient “competition” toward cancer cells. They also reported that miR-122 derived from breast cancer cells could mediate the suppression of glucose uptake in lung fibroblasts and astrocytes (39).

### Effect of Exosomal miRNAs Released by CAFs on Cancer Cells

Accumulating evidence has indicated that exosomal miRNAs secreted from the CAFs directly mediate the cell–cell interaction and play a crucial role in promoting tumor development.

One of the typical exosomal miRNAs is miR-21, which has been widely recognized as an oncogene promoting cell proliferation, migration, and invasiveness. Mechanically, it functions via targeting a number of tumor suppressor genes including p53 and PTEN (56) and enhancing the expression of VEGF, which thus promotes angiogenesis (79). Recently, more studies have explored the function of encapsulated miR-21 in TME associated with the crosstalk between cancer cells and CAFs. An *in vivo* study showed that miR-21-overexpressing fibroblasts led to the progression of CRC and increased liver metastases. Other miRNAs were also transferred into CRC cells via CAFs-derived exosomes and altered the phenotype and biological behavior of CRC cells (53). Fibroblasts with a high level of miR-21 could also protect CRC cells from oxaliplatin-induced apoptosis and increase their proliferation and epithelial invasiveness via a conditioned medium containing ectopic stromal miR-21 (52). MiR-21 released by CAFs was also related to the cisplatin resistance in GC cells by suppressing cell apoptosis and enhancing the activation of the PI3K/Akt signaling pathway resulting from the downregulation of PTEN (56). In ovarian cancer, the exosomal transfer of miR21 isolated from cancer-associated adipocytes and fibroblasts (CAFs) conferred paclitaxel resistance through targeting apoptotic protease activating factor 1 (APAF1) (63). Reciprocally, miR-21 could, in turn, promote CAF formation and activation (62).

CAF-derived ectopic stromal miRNAs regulate the tumor-promoting effects through various cytokines and signaling pathways. One of the most significant effects is promoting tumor proliferation. CAFs regulate tumor proliferation by transferring exosomal miR-34a-5p to neighboring tumor cells in in oral squamous cell carcinoma (OSCC) (64). A downregulation of miR-335 in hepatocellular carcinoma (HCC) fibroblasts/stellate cells was observed, promoting cell proliferation and invasiveness. Conversely, the upregulation led to the regression of cancer (58). Shen et al. demonstrated that the overexpression of miR-7 in CAFs led to the downregulation of RAS-association domain family 2 (RASSF2), a member of Ras-association domain family of protein, which dramatically decreased the secretion of protease-activated receptor-4 (PAR-4) from CAFs and then enhanced the proliferation and migration of the co-cultured cancer cells (59). Josson et al. primarily demonstrated the specific role of miR-409-3p/-5p in prostate cancer biology by facilitating tumorigenesis, EMT, and bone metastasis (66). Next, they found that the ectopic expression of miR-409 in NFs conferred a cancer-associated stroma-like phenotype and led to the release of exosomal miR-409, thus triggering tumor induction, EMT, and stemness of the epithelial cancer cells *in vitro* and *in vivo*. The repression of tumor suppressor genes, such as Ras suppressor 1 and stromal antigen 2, led to this effect (67).

Various exosomal miRNAs and signaling pathways also participate in invasion and metastasis, another typical hallmark of cancer cells which is generally considered a multistep process (76). MiR-15a was identified as a downregulated miRNA that could suppress the migration of cholangiocarcinoma (CCA) cells in CCA-associated fibroblasts, with higher expression of its target gene plasminogen activator inhibitor 2 (PAI 2) (51). Another significantly decreased miRNA was exosomal miR-320a. It binds to its direct downstream target pre-B cell leukemia homeobox 3 (PBX3) and thus suppresses the activation of the MAPK pathway, inhibits the expression of cyclin-dependent protein kinases 2(CDK2) and MMP2 and inhibits EMT. Here, both CAFs and corresponding para-cancer fibroblasts release exosomes and transfer miRNA to HCC cells (57). Loss of exosomal miR-148b from CAFs also promotes endometrial cancer cell invasion and cancer metastasis via binding to DNA methyltransferase 1 (DNMT1), as shown in a recent study. EMT also plays a role in this process (55). High expression levels of miR-9 in primary triple-negative breast CAFs could improve the migration and invasion capabilities by modulating its direct target E-cadherin, a key cell-to-cell adhesion molecule the loss of which was defined as the best-characterized alteration improving metastasis (76) and NF biological behavior (50). MiR-21, miR-378e, and miR-143 also promoted a significantly increased capacity to form mammospheres, anchorage-independent cell growth, and stemness and EMT phenotype of breast cancer cells, and thus dictated an aggressive phenotype in breast cancer (49). Exosomal miR-1228 from CAFs promotes the cell migration and invasion of osteosarcoma by directly downregulating endogenous suppressor of cancer cell invasion (SCAI) mRNA and protein levels (65).

Drug resistance is an important factor affecting tumor prognosis. Accumulating studies have tried to uncover the mechanism. However, when talking about the role of exosomal miRNAs released by CAFs, the underlying mechanism remains largely unclear. Many studies have suggested that CAFs are associated with drug resistance in various types of tumors by regulating diverse cytokines, intracellular signaling pathways, and proteins, or via other mechanisms. A noteworthy feature is that CAFs exposed to gemcitabine significantly increase the release of exosomes with increased chemoresistance-inducing factor Snail and a marked increase in miR-146a expression. When transferred into recipient epithelial cells, they promote cell proliferation. This endows cancer cells with drug resistance (80). Another study proposed that CAF-secreted exosomes would contribute to chemoresistance in CRC through priming cancer stem cells (81). Li et al. then proposed that miR-1 mediated the paracrine effect of CAFs on lung cancer cell proliferation and chemoresistance via the CXCR4-mediated signaling pathway, which involved NF-κB and Bcl-xL (61). Exosomal miR-21 released by TAMs or stroma also led to the cisplatin resistance in GC by down-regulating PTEN (56) and paclitaxel resistance in ovarian cancer through targeting APAF1 (63). Additionally, CAF-derived exosomal miR-196a was found to confer cisplatin resistance in head and neck cancer (HNC) by targeting cyclin-dependent kinase inhibitor 1B (CDKN1B) and inhibitor of growth 5 (ING5) (60).

Interestingly, a study showed that the exosomal miR-9 in breast cancer could, in turn, convert NFs into CAFs (50).

Another influence exerted by CAF-derived exosomal miRNAs is on autophagy. Autophagy is a double-edged sword in tumor development, which has gained great significance in the last few years. Despite being an emerging field replete with major unanswered questions, a large number of studies investigated the important features of autophagy in cancer. “The reverse Warburg effect” was primarily proposed as a tumor metabolism autophagic interstitial model by Pavlides et al. (82). It was presumed that the autophagy of CAFs might be involved in tumor energy acquisition. For instance, miR-105 secreted by cancer cells were capable of inducing a metabolic program in CAFs (38). Further, a recent study showed that miR-31 up-regulated in CAFs inhibited autophagy in colorectal CAFs and significantly affected CRC cell behaviors, including proliferation, invasion, apoptosis, and radiosensitivity. This function took effect via repressing the expression of the autophagy-related genes Beclin-1, autophagy-related (ATG), damage-regulated autophagy modulator (DRAM), and LC3 (54).

## Crosstalk Between Tumor Cells and TAMs Via Exosomal miRNAs

### TAMs

Compelling evidence has emerged for the tumor-promoting role of macrophages in TME over the past decade. TAMs are the predominant leukocytes infiltrating solid tumors. They are largely derived from circulating monocytes (2) and are typically of M2-like phenotype (3). M1 macrophages act as soldiers, producing high amounts of inflammatory cytokines (IL-12 and TNF-α) and fighting against tumors. M2 macrophages are activated by Th2 cytokines (IL-4, IL-10, and IL-13), and an excess of M2 macrophages can lead to severe immune suppression, thus shaping a tumor-facilitating microenvironment. TAMs have been found to favor malignant progression by suppressing antitumor immunity, stimulating angiogenesis, enhancing tumor cell proliferation (83), angiogenesis, and metastasis (84, 85). They are also competent effector cells remodeling TME, including both extracellular matrix and other immune cells (86). Therefore, the transformation and recruitment of TAMs at the tumor site play a pivotal role. Next, we would focus on the mechanisms and functions of crosstalk between tumor cells and TAMs in TME mediated by miRNAs (Table 2).

**Table 2:** Exosomal miRNAs mediating the communication between tumor cells and TAMs.

**Origin**	**miRNAs**	**Donor (Markers)**	**Receptor (Markers)**	**Target and mechanism**	**Function**	**References**
Tumor cells	miR-25-3p, miR-130b-3p, miR-425-5p	CRC	TAMs (CD206)	PTEN/PI3K/Akt signaling pathway CXCL12/CXCR4 axis	Inducing M2 polarization of tumor-associated macrophages	(87)
	miR-145	CRC	TAMs (CD11b, CD68, CD206)	Downregulating histone deacetylase 11	Inducing polarization of tumor-associated macrophages	(88)
	miR-1246	CRC	TAMs (CD206, CD163)	TGF-β	Reprograming macrophages; favoring anti-inflammatory immunosuppression	(89)
	miR-21-3p, miR-125 b-5p, miR-181 d-5p	EOC	TAMs (CD206, CD163, IL-10)	SOCS4/5/STAT3 pathway	Inducing polarization of TAMs	(90)
	miR-23a-3p	HCC	TAMs (CD 206, IL-10)	PI3K-AKT pathway	Inducing high expression of PD-L1	(91)
	miR-146a-5p	HCC	TAMs (CD 206, CD 11b)	NF-κB signaling pathway and secreting various pro-inflammatory factors (IL-6, IL-8)	Reversed T cell exhaustion, and delayed HCC progression	(92)
	miR-21	HNSCC	TAMs (MRC1, CD163, and IL10)	Snail	Inducing M2-like polarization of CD14^+^ human monocytes	(93)
	miR-103a	LAC	TAMs (CD 206)	PTEN/Akt/STAT3	Inducing polarization of tumor-associated macrophages; progression and angiogenesis	(94)
	miR-25-3p, miR-92a-3p	Liposarcoma	TAMs (CD 68)	TLR7/8	Favoring secretion of pro-inflammatory cytokine IL6	(95)
	miR-222-3p	OC	TAMs (CD 68, iNOS)	SOCS3 and JAK/STAT signaling pathway	Inducing polarization of tumor-associated macrophages, angiogenesis, and lymphangiogenesis	(96)
	miR-301a-3p	PDAC	TAMs (CD206, CD163, IL10, TGFβ, and arginase-1)	PTEN/PI3Kγ signaling pathway	Inducing polarization of tumor-associated macrophages, migration, invasion, and EMT	(97)
TAMs	miR-5100	TAMs (CD 11b)	BC	CXCL12/CXCR4 axis	Inhibiting the invasion, migration and EMT of breast cancer cells.	(98)
	miR-21-5p, miR-155-5p	TAMs (CD 68, CD 163, CD206)	CRC	BRG1	Promoting cell migration and invasion	(99)
	miR-21	TAMs (CD11b, CD206, CD86)	GC	PTEN/PI3K/Akt signaling pathway	Inducing cisplatin resistance	(56)
	miR-125a, miR-125b	TAMs (–)	HCC	CD90	Favoring tumorigenesis and progression	(19)
	miR-155-5p	TAMs (CD 206)	Smooth muscle cells	GREM1	Promoting proliferation, migration, and Intracranial aneurysm formation	(100)
	miR-365	TAMs (F4/80)	PDAC	Upregulating the triphosphonucleotide pool in cancer cells; inducing the enzyme cytidine deaminase	Inducing gemcitabine resistance	(101)

### Role of Exosomal miRNAs Released by Cancer Cells on TAMs

The bidirectional interplay between cancer cells and TAMs is crucial and is largely mediated by bioactive molecules transmitted via exosomes, mainly miRNAs. As mentioned earlier, macrophages are flexible cells and their phenotype switch primarily depends on the various stimuli from the milieu. One of the most significant roles of the communication between cancer cells and TAMs mediated by TEX-derived miRNAs is promoting the transformation of TAMs. (102) established that significantly increased miR-222-3p packaged in epithelial ovarian cancer (EOC)-secreted exosomes induced the polarization of TAMs via targeting the SOCS3 gene (96), a negative feedback regulator of the JAK/STAT signaling pathway. They also verified that miR-222-3p promoted angiogenesis and lymphangiogenesis in TME (96). The snail-overexpressing cancer cells could release miR-21-abundant exosomes, which were engulfed by CD14^+^ human monocytes, suppressing the expression of M1 markers and increasing that of M2 markers (93). miR-1246, a kind of miRNA enriched in exosomes derived from mutant p53 cancers, is capable of reprograming macrophages to a tumor-supportive and anti-inflammatory state, increasing the activity of TGF-β and favoring anti-inflammatory immunosuppression (89). In addition, Shinohara et al. demonstrated that CRC cells positively secreted miR-145 via exosomes. They were taken up by macrophage-like cells and polarized them into M2-like phenotype through the downregulation of histone deacetylase 11 (88). A recent study has showed that several upregulated exosomal miRNAs (miR-25-3p, miR-130b-3p, miR-425-5p) in CRC cells, could induce M2 polarization of macrophages by regulating PTEN via CXCL12/CXCR4 axis.

Hypoxia is a common state in various types of cancers and is commonly implicated in the establishment of an immunosuppressive niche. Accumulating evidence elucidated that hypoxia would induce genetic and proteomic changes in cancer cells, as well as facilitate the exosome secretion (103, 104). A study revealed that epithelial ovarian cancer (EOC) cell–derived exosomes induced by hypoxia delivered higher levels of miR-21-3p, miR-125 b-5p, and miR-181 d-5p to macrophages. They induced the polarization of M2 macrophages via regulating the SOCS4/5/STAT3 pathway and promoted EOC cell proliferation and migration in a feedback loop (90). Hypoxia also induces epithelial ovarian cancer cells to secrete exosomes capsuling miRNA-940. The internalization by unpolarized macrophages stimulated the polarization toward a TAM-like phenotype (105). These results highlighted the function of hypoxic TME–derived TEXs in enhancing M2-like polarization and stimulating tumor progression.

Further, the TAM-derived exosomes also regulate various biological behaviors of tumor cells. Casadei et al. proposed that miR-25-3p and miR-92a-3p secreted by liposarcoma cells favored the secretion of pro-inflammatory cytokine IL6 from TAMs in a TLR7/8-dependent manner, which in turn promoted liposarcoma cell proliferation, invasion, and metastasis (95). Meanwhile, hypoxia also drove the release of exosomes in lung cancers, which increased M2-type polarization via miR-103a transfer. Exosomal miR-103a decreased PTEN levels and increased the activation of Akt and STAT3 as well as the expression of several immunosuppressive and pro-angiogenic factors, further enhancing cancer progression and tumor angiogenesis (94). miR-150 delivered by exosomes also promoted tumorigenesis by upregulating VEGF in TAMs, while the neutralization of miR-150 attenuated angiogenesis and tumor development (106). Besides the proliferation of tumor cells that initiates cancer and the angiogenesis that maintains the growth of tumors, exosomal miRNAs also facilitate invasion into surrounding normal tissues and metastasis to local and distant sites. Wang et al. (107) elucidated that hypoxic exosomes derived from pancreatic cancer cells activated macrophages to the M2 phenotype in a hypoxia inducible factor (HIF) 1a- or HIF2a-dependent manner via activation of the PTEN/PI3Kγ signaling pathway. The formation of TAMs then facilitates the migration, invasion, and EMT of pancreatic cancer cells, thus promoting metastasis (97).

### Effect of Exosomal miRNAs Released by TAMs on Cancer Cells

Reciprocally, exosomes derived from TAMs play an essential role in shuttling between cancer cells and macrophages via secreting miRNAs, facilitate specific tumor biological behavior, and induce the acquisition of a specific biological phenotype of recipient cells. A large number of studies focused on the immune-suppressive function and tumor-promoting role of TAMs via diverse cytokines and intracellular signaling pathways. However, the function mediated by exosomes released from TAMs requires more exploration. Lan et al. found that M2 macrophage–derived exosomes (MDEs) displayed a high expression level of miR-21-5p and miR-155-5p, which bound to the Brahma related gene 1(BRG1)-coding sequence after being absorbed by CRC cells and downregulated the expression of BRG1, thus promoting cell migration and invasion in colon cancer (99). BRG1 has been identified as a core motor of SWI/SNF, which decreases in tumor tissues, thus promoting CRC metastasis. In addition, miR-155-5p also could promote intracranial aneurysm (IA) formation, and in turn, promote TAM activation and infiltration (100). Chemoresistance is another obstacle affecting clinical therapy efficacy. A study showed that MDE-derived miR-21, which participates in various processes of tumor progression, conferred cisplatin resistance in GC cells by suppressing cell apoptosis and enhancing the activation of PI3K/AKT signaling pathway via the downregulation of PTEN (56). A study pointed out that the transfer of miR-365 in MDE significantly decreased the sensitivity of pancreatic ductal adenocarcinoma (PDAC) cells to gemcitabine. The upregulation of the triphosphonucleotide pool in cancer cells and the induction of the enzyme cytidine deaminase were two major changes in this case (101). Intriguingly, (19) reported that exosomes derived from TAMs with significantly low levels of miR-125a and miR-125b orchestrated stem cell properties in hepatocellular carcinoma by targeting CD90, thus favoring the tumorigenesis and progression of HCC. Recently, Yue et al. (98) constructed a PGRN^−/−^ mouse breast cancer xenograft model and found PGRN^−/−^ TAMs-derived miR-5100 was up-regulated inhibited lung metastasis of breast cancer.

Studies also demonstrated that exosomal miRNAs derived from TAMs in TME were also capable of regulating the immune response. For instance, a study showed that exosomes enriched with miR-29a-3p and miR-21-5p released by TAMs could be taken up by CD4^+^ T cells. The transfected miRNAs directly suppressed the STAT3 signaling pathway and led to a significantly higher Treg/Th17 cell ratio *in situ* and in metastatic peritoneal tissues in EOC (108). The imbalance of Treg/Th17 has been observed in many diseases, and the regulated milieu exhibits an immune-suppressive profile (109, 110). Meanwhile, according to existing literature, HCC cells secreted miR-23a-3p can target M2 type TAMs. MiR-23a-3p inhibited the expression level of PTEN, inducing high expression of PD-L1 via PI3K-AKT pathway (91). HCC derived exosomeal miR-146a-5p also could remodel macrophages by NF-κB signaling pathway and secreting various pro-inflammatory factors (IL-6, IL-8) (92). However, the immunomodulation induced by exosomal miRNAs is complex and dynamic, the related mechanisms need to be further clarified.

## Crosstalk Between TAMs and CAFs Via Exosomal miRNAs

CAFs can influence the function of immune cells in TME by producing growth factors, cytokines, and exosomes, and exosomal miRNA of CAFs delivery is a novel way. For instance, hypoxia and nutrient deficiency in TME, resulted in an inflammatory microenvironment formation. The secretion of inflammatory mediators changed the functions and status of various immune cells in TME and activated NFs to CAFs. During hypoxia, activated CAFs secreted a high level of miR-21 to promote tumor progression and monocyte recruitment. CAFs-derived miR-21 taken up by monocytes could induce M2 polarization of TAMs. CAF-educated M2 macrophages exerted their immunosuppressive roles via the PD-1 axis (111). In turn, MDE-derived miR-21 stimulate the phenotypic transformation of CAFs via PTEN/PI3K/Akt signaling pathway (87, 112). Hence, as a novel way mediating cellular interactions that involve CAFs and TAMs, these studies provided a new insight into the complex regulation of tumor microenvironment.

## Engineering Exosomal miRNAs as Novel Therapy Biomarkers

Exosomes are biological nanocarriers which could be taken part in various treatment of various diseases, miRNA play an important role. Modification of exosomes can deliver tumor-suppressive miRNAs to tumor cells via engineering technology. Engineered exosomes were used to deliver an anticancer drug 5-FU and miR-21 inhibitor (miR-21i) to colon cancer cells via electroporation, which effectively reversed drug resistance and reduced tumor proliferation (113). Kim et al. produced a glioblastoma-targeting carrier with the T7 peptide and exosomes. Then AMO-21 (miRNA oligonucleotides against miR-21) was carried into the exosomes by electroporation. T7-exo delivered AMO-21 decreased the miR-21expression, inducing the expression of PDCD4 and PTEN in glioblastoma (114). In addition, exosomal miRNAs are recognized as promising biomarkers to early diagnose cancer. As exosomal miRNAs can be distributed to bio-fluids, including urine, plasma, and cerebrospinal fluid, is a non-invasive way to obtain exact information about tumor status (115). A lastest study has reported that Zhou et al. (116) designed a 3D microfluidic chip with three exquisitely engineered virus-mimicking fusogenic vesicles, which can multiple exosomal miRNAs (such as miR-451a, miR-21, and miR-10b) (116). Therefore, the role of exosomal miRNAs is important by using bioengineering technology and developing safe and effective new materials.

## Discussion

Increasing lines of research demonstrate that exosomal miRNAs play a critical role in different kinds of cancer via mediating the intercellular communication between tumor and stromal cells. Further, these bidirectional signal transductions also reshape TME. Thus, the exosomal crosstalk between tumor cells, CAFs, and TAMs largely motivates the development of tumors. As illustrated by recent experimental data, exosomes released by diverse cells under different circumstances possess specific non-coding RNA profiles. As a credible way to isolate cancer-associated molecules that provide pathological information, bioactive molecules are more stable (relatively hard to be degraded) with a higher concentration in exosomes compared with circulating RNAs, which makes exosomes a potential non-invasive target for diagnosis. Therefore, circulating exosomes help in both diagnosis and well-designed, targeted therapeutic schemes. An efficient way is to block the production and the uptake of exosomes. Antibodies aiming to reduce the secretion of tumor exosomes help reduce tumor progression and metastasis. Exosomal miRNAs, as key messengers, were also identified as therapeutic targets to block recruitment, polarization, activation, and release of TAMs and CAFs, which helps abrogate tumor development. Gene therapy via engineering miRNAs may be an effective strategy. Other cargo molecules also play a role and have gained attention due to their potential availability as detected markers and also therapeutic targets. Moreover, single therapy inhibiting CAFs or TAMs only might not be effective. A therapy targeting specific cell–cell crosstalk and reducing the number of CAFs and TAMs might be an effective approach.

However, the field of exosomes still faces a plethora of challenges and deserves further study. First, our understanding of molecular mechanisms underlying EVs biogenesis is still incomplete, which need to be further studied. Further, the biological mechanism which selecting and guiding specific miRNAs into exosomes is still not entirely understood, and more studies are required for exploring both the underlying mechanisms and also how they altered at different circumstances and different stage. Moreover, a standard method to extract and purify exosomes for a clinically relevant application has yet to be established, which limits the potential use of exosomal miRNAs as diagnostic and prognostic markers. Also, the miRNA content of plasma in exosome fractions from patient blood samples was low due to insufficient pathological diagnosis and extraction technology. A quantitative assessment proved that even the most abundant miRNAs were far less than one molecule per exosome on average. Hence, the aim was to isolate pure groups of specific subtypes of exosomes and figure out their further response, so that these diverse subtypes could be selected for clinical use. Further, it is worthwhile establishing a platform that exhibits different types of miRNAs in exosomes and their functions in TME, so that exosomal miRNAs can be detected as biomarkers. Moreover, compared with other nanoparticles, exosomes have many advantages: low immunogenicity and toxicity, excellent biocompatibility, increased stability, and targetability (117). Thus, therapeutics based on exosomes could be an emerging approach. However, further exploration is still needed. Significant progress has been made in describing the role of exosomes in TME, and it might be a promising and exciting platform for tumor therapy.

## Author Contributions

TS drafted the manuscript. PZ and FZ revised the manuscript. SZ provided direction and revised the manuscript. All authors read and approved the final manuscript.

## Conflict of Interest

The authors declare that the research was conducted in the absence of any commercial or financial relationships that could be construed as a potential conflict of interest.

## Abbreviations

CRC: Colorectal cancer
CLL: Chronic lymphocytic leukemia
HCC: Hepatocellular Carcinoma
GC: Gastric cancer
BC: Breast cancer
LAC: Lung adenocarcinoma
HNC: Head and neck cancer
CCA: Cholangiocarcinoma
OC: Ovarian cancer
OS: Osteosarcoma
EMC: endometrial cancer
PC: Prostate cancer
PDAC: Pancreatic cancer
OSCC: oral squamous cell carcinoma
α-SMA: α-smooth actin
PDGFR-β: platelet-derived growth factor receptor-β.
